# Distal Femoral Shortening Osteotomy for Severe Knee Flexion Contracture and Crouch Gait in Cerebral Palsy

**DOI:** 10.3390/jcm8091354

**Published:** 2019-09-01

**Authors:** Hoon Park, Byoung Kyu Park, Kun-Bo Park, Sharkawy Wagih Abdel-Baki, Isaac Rhee, Chan Woo Kim, Hyun Woo Kim

**Affiliations:** 1 Department of Orthopaedic Surgery, Gangnam Severance Hospital, Yonsei University College of Medicine, 211 Eonju-ro, Gangnam-gu, Seoul 06273, Korea; 2 Department of Orthopaedic Surgery, Inje University Haeundae Paik Hospital, Busan 48108, Korea; 3 Division of Pediatric Orthopaedic Surgery, Severance Children’s Hospital, Yonsei University College of Medicine, 50-1 Yonsei-ro, Seodaemun-gu, Seoul 03722, Korea; 4 Department of Orthopaedic Surgery, Aswan University Hospital, Aswan University Faculty of Medicine, Aswan 81528, Egypt; 5 Medical course, University of Melbourne Melbourne Medical School, Melbourne 3010, Australia

**Keywords:** distal femoral shortening osteotomy, patellar tendon advancement, severe knee flexion contracture, crouch gait, cerebral palsy

## Abstract

Although there have been advancements of surgical techniques to correct gait abnormalities seen in patients with cerebral palsy, the crouch gait remains one of the most difficult problems to treat. The purpose of this retrospective study was to examine our results of distal femoral shortening osteotomy (DFSO) and patellar tendon advancement (PTA), performed in patients with crouch gait associated with severe knee flexion contracture. A total of 33 patients with a mean fixed knee contracture of 38° were included in the study. The mean age at the time of surgery was 12.2 years and the mean follow-up was 26.9 months. The measurements of clinical, radiological, and gait parameters were performed before and after surgery. The mean degrees of knee flexion contracture, Koshino index of patella height, and Gait Deviation Index were found to be significantly improved at the time of final follow-up. The maximum knee extension during the stance phase improved by an average of 25°, and the range of knee motion during gait increased postoperatively. On the other hand, the mean anterior pelvic tilt increased by 9.9°. Also, the maximum knee flexion during the swing phase decreased and the timing of peak knee flexion was observed to be delayed. We conclude that combined procedure of DFSO and PTA is an effective and safe surgical method for treating severe knee flexion contracture and crouch gait. However, the surgeons should be aware of the development of increased anterior pelvic tilt and stiff knee gait after the index operation.

## 1. Introduction

Crouch gait is characterized by excessive knee flexion and ankle dorsiflexion during the stance phase of gait, and it leads to an ineffective ankle plantarflexion/knee extension couple [1,2,3]. Traditionally, crouch gait seen in patients with cerebral palsy (CP) has often been an iatrogenic consequence of isolated gastrocsoleus lengthening in a jump gait pattern when the other levels of gait deviations have not been addressed properly [4,5,6]. The pathophysiology of this gait abnormality is multifactorial; however, any conditions leading to lever arm dysfunction may be attributed to the development of a crouch gait [7]. Crouch gait remains one of the most difficult problems to treat [2,5], and it may result in anterior knee pain and patellar instability due to the high patellar tendon forces exerted [8,9].

Hamstring lengthening is a commonly used method to correct hamstring tightness and the resultant increased knee flexion during walking [2,10,11,12]. However, outcomes of hamstring lengthening were variable and the procedure were not found to be effective in the treatment of a fixed knee flexion contracture [2,12,13]. Distal femoral extension osteotomy (DFEO) has been described as an alternative to treat fixed knee contractures [14,15,16,17,18,19,20]. Also, patellar tendon advancement (PTA) has often been coupled with DFEO to rectify concomitant patella alta and quadriceps insufficiency associated with long-standing crouch gait [7,21,22].

**Figure 1:**
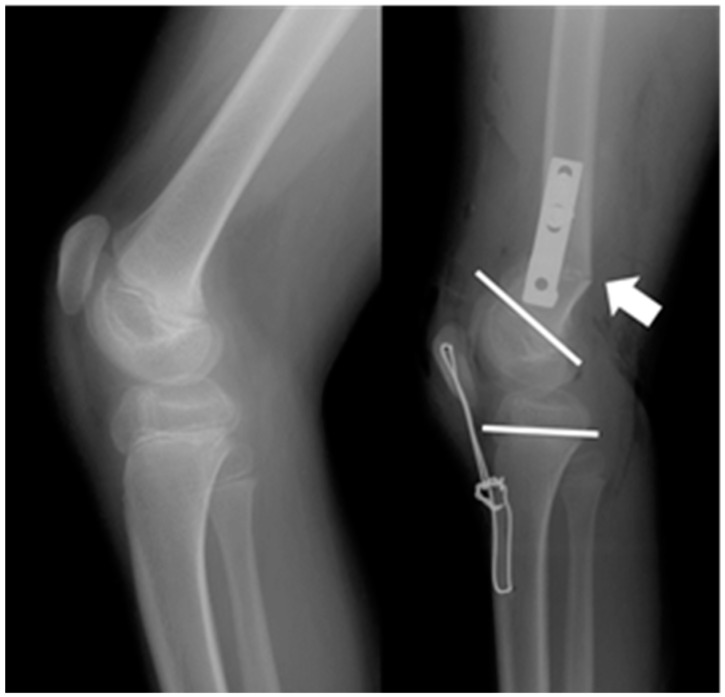
Preoperative and postoperative radiographs of a patient whose preoperative knee flexion contracture measured 43°. After resection of a wedge-shaped bone via a distal femoral extension osteotomy, significant angular deformity developed at the distal femur. Difficulty in securing fixation with a plate may be encountered and the weight-bearing surface of the distal femur may shift to the posterior condyles during the mid-stance phase of the gait.

However, simple femoral extension osteotomy can lead to several problems in patients with severe knee flexion contracture. Posterior bony prominences can develop after correction of the deformity and may cause neurovascular compromise, whilst the resultant hamstrings stretch may further increase the risk of postoperative nerve palsy [15,23,24]. Moreover, there is a possibility that the hyperextension, created at the osteotomy site, may cause tibio-femoral joint incongruity and eccentric articular cartilage damage over time (Figure 1). A possible alternative to minimize the above-mentioned problems would be distal femoral shortening osteotomy (DFSO); however, there is a paucity of the literature on this technique and its outcomes in the management of CP [25,26].

During the last fifteen years, it has been our practice that we conducted “shortening” rather than “extension” femoral osteotomy for the ambulant whose severe crouch gait is associated with fixed knee flexion contracture of more than 30 degrees. We had decided this was the appropriate course of action to avoid any neurovascular complications, to maintain congruity of the knee joint and to achieve sufficient correction of the deformity. In this retrospective cohort study, we examined our results of combined DFSO and PTA performed as part of a single-event multilevel surgery. To the best of our knowledge, this is the first study to investigate and assess the overall results of the index operation, especially from the view point of three-dimensional gait analysis.

## 2. Experimental Section

### 2.1. Subjects

This retrospective study was approved by our Institutional Review Board (IRB No. 4-2013-0282). We reviewed the medical records, plain radiographs, and reports of three-dimensional gait analysis of patients with CP who were treated with DFSO and PTA between March 2004 and December 2016. The indication of the index operation was a knee flexion contracture of more than 30 degrees examined under general anesthesia. The inclusion criteria for the study were as follows: (1) a primary diagnosis of spastic diplegia; (2) an ability to walk defined by the Gross Motor Function Classification System (GMFCS), level I, II, or III [27]; (3) a minimum postoperative follow-up period of 2 years; and (4) performance of pre- and postoperative three-dimensional gait analysis within 3 months before surgery and 2 years or more after surgery.

We identified 40 consecutive patients who met the inclusion criteria. Three patients were lost to radiological follow-up and four patients were excluded since there were no reports of either pre- or postoperative gait analysis available for review. Thus, a total of 66 limbs in 33 patients were included in the present study. There were 20 males and 13 females. The mean age at the time of surgery was 12.2 years (range, 6.0 to 16.6 years), and the mean follow-up period was 26.9 months (range, 24.0 to 47.0 months). Nineteen patients were in GMFCS level II, and 14 patients level III.

### 2.2. Surgical Technique

**Figure 2:**
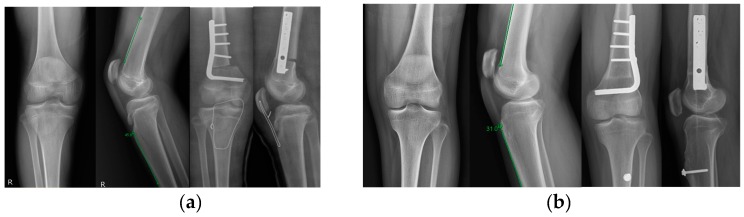
Preoperative and postoperative anteroposterior and lateral radiographs of the knee joints showing distal femoral shortening osteotomy fixed with a blade plate and correction of patella alta with patella tendon shortening and tension-band wiring (**a**) or tibial tubercle advancement (**b**), respectively.

All procedures were performed by a single surgeon (H.W.K.) (Figure 2). In supine position, a lateral approach to the distal femur was used and the supracondylar area was exposed. The anticipated amount of femoral shortening was planned with the consideration of the degrees of knee flexion contracture. The length of the rectangular-shaped bony resection was adjusted so that the bilateral popliteal angle measured about 30–35° and the knee achieved full extension when the hip was extended after fixation with a plate. If the knee flexion contracture remained after this first trial of femoral shortening, further shortening or minimal tilting of the distal fragment was performed; for 5 patients in order to straighten the knee, even after considerable femoral shortening, we slightly tilted the distal fragment into minimal extension. The mean femoral shortening was 2.8 cm (range, 2 to 4 cm). Any increased femoral anteversion of more than 30 degrees was corrected simultaneously. In 23 patients a 90° angled blade plate (DePuy Synthes, West Chester, PA, USA) was used for fixation, and a locking plate (DePuy Synthes, West Chester, PA, USA) in the remaining 10 patients.

For PTA, an anterior longitudinal or slightly oblique incision was made in line with the patellar tendon. If the tibial growth plate was still open, a T-shaped periosteal incision was made just distal to the tibial tubercle and the medial and lateral periosteal flaps were then elevated. The patellar tendon was split longitudinally in the sagittal plane, and the dorsal half of the tendon was separated from the tibial tubercle whilst the ventral half was not divided from their respective attachment sites. Using a right-angled clamp, the dorsal half of the patellar tendon was advanced distally until the superior pole of the patella was located at the distal femoral physis or slightly distal to it. Whilst maintaining tension on the patellar by the dorsal advancement, the ventral half was shortened by 2–3 cm by using an overlapping suture. The elevated periosteal flaps were then sewn over the distal region of the dorsal half of the patellar tendon that had been maintaining the advancement. The proximal region of the dorsal half of the patellar tendon was then sutured to the previously overlapped ventral half of the patellar tendon. A tension-band wire was placed transversely through the patella and the proximal tibia to protect the repair. If the physis was closed, a small rectangular-shaped tibial tubercle bone block with the patellar tendon attached was created. A new recipient site for the tibial bone block was determined with the same manner as described earlier under the intraoperative fluoroscopic guidance. A distally advanced tibial tubercle block was secured with a compression screw.

Details on previous surgeries and simultaneous procedures at the time of the index operation are summarized in Table 1. Nineteen (57.6%) of the 33 subjects had had previous surgical interventions elsewhere. Among these, seventeen (89.4%) had undergone hamstrings lengthening and 16 (84.2%) had undergone tendo-Achilles lengthening. Twenty-nine (87.8%) patients had concurrent procedures in addition to the index operation but no patients had hamstrings lengthening in the same surgical setting. Eleven patients underwent plantarflexor release and lateral column lengthening with or without medial column reconstruction in order to correct a planovalgus foot deformity. Four patients had DFSO and PTA as an isolated procedure without doing additional surgeries.

The postoperative management was identical for all patients. An above-the-knee cast was applied for 4 weeks. After removal of a cast, comprehensive physiotherapy including protected and progressive range of motion exercise of the joint and the weight bearing was begun. A rigorous strengthening exercise for the quadriceps and the triceps was encouraged.

### 2.3. Clinical, Radiological, and Gait Parameters

Thorough physical examinations were conducted on the dates of preoperative and final gait analysis. These included the measurements of range of motion of a joint, degrees of fixed knee flexion contracture, and the determination of hamstrings contracture or tightness as demonstrated by the popliteal angle.

Pre- and postoperative standing radiographs of the lower extremities were used for radiological evaluations. Patellar height was evaluated according to the method described by Koshino and Sugimoto [28,29]; a ratio was calculated between the midpoints of the femoral and tibial epiphyseal lines or their scar and the midpoint of the long axis of the patella. The Koshino index was reported as a normative (z-score) measure calculated relative to that of a healthy child based on the knee flexion angle in a lateral radiograph [28]. Any progressive remodeling occurring at the original osteotomy site was examined by comparing the physeal posterior distal femoral angles (pPDFA) before and after surgery. The pPDFA is formed by intersecting the femoral anatomic axis and distal femoral physeal line in the sagittal plane [23].

Kinematic and kinetic analyses were performed with the use of the VICON Motion System (Oxford Metrics, Oxford, England) with six infrared cameras and a Helen Hayes marker set. Ground reaction force data were gathered with the aid of multiple force platforms (Advanced Mechanical Technology Inc., Watertown, MA, USA). The data from the force plate were low-pass filtered using a Butterworth filter. All subjects were asked to walk barefoot at a self-selected speed along a 15-m walkway with the markers in place. The normal range for kinematic data was defined as two standard deviations from the mean. Force plates under the path recorded the ground reaction forces during walking trials and joint moments were expressed as internal moments to counter the ground reaction forces. Kinematic and kinetic data from successful trials were averaged and used for statistical analysis. Fourteen patients classified as having walking ability of GMFCS level III used a walker during the study, and only the kinematic data were obtained in those patients. The reference values for the kinematic data was previously collected from 72 normal pediatric volunteers from our previous study [30].

The pre- and postoperative temporospatial, kinematic, and kinetic variables were compared to examine the effects of the index operation on gait changes in the whole cohort. The Gait Deviation Index (GDI) score [31] was calculated to determine the gait functional improvement after surgery. GDI compares nine kinematic variables of a subject’s gait against those of a control group; this requires kinematic data from the pelvis and hip in all three planes, the knee and ankle in the sagittal plane, and foot progression [32]. A GDI score >100 denotes a non-pathological gait, and each 10-point decrement below 100 represents 1 standard deviation from normal kinematics [32].

Furthermore, we performed a subgroup analysis to investigate whether simultaneous correction of a planovalgus foot deformity with a plantarflexor release may affect the surgical outcomes. All patients were divided into two sub-groups; Group 1 consisted of 22 patients having no foot surgery and Group 2 was comprised of 11 patients who underwent simultaneous lateral column lengthening with or without medial column reconstruction. Pre- and postoperative gait parameters were compared between the groups.

### 2.4. Statistical Analyses

Statistical analyses were performed using SAS (Version 9.4, SAS Inc., Cary, NC, USA). Gait and radiological data were analyzed using a linear mixed model, allowing for fixed effects of time from pre- to postoperatively as well as random effects of bilaterality. In order to compare pre- and postoperative variables between the groups, we used a linear mixed model with the surgical treatment as the fixed effect and the bilaterality as the random effect. In all analyses, *p* < 0.05 was considered significant.

## 3. Results

There were no major postoperative complications such as nonunion or neurological compromise, and minor complications included 2 superficial wound infections and 3 heel sores. The GMFCS level did not change after surgery in any of the patients. The mean knee flexion contracture improved from 38° ± 6° preoperatively to 12° ± 7° postoperatively (*p* < 0.001). The mean popliteal angle decreased significantly by 22° postoperatively (*p* < 0.001).

The mean pre- and postoperative Koshino indices were 1.56 ± 0.17 and 1.24 ± 0.17, respectively and this change was statistically significant (*p* < 0.001). The z-score of the Koshino index also decreased (*p* < 0.001) (Table 2). The mean pPDFAs before and after surgery were 86.7° ± 3.6° and 88.1° ± 4.6°, respectively. Although there was a significant change after surgery, this difference was only 1.4°.

There were no postoperative changes in the temporospatial parameters such as in walking speed, step length, and cadence (Table 2). The mean GDI score significantly improved from 69.5 (range, 54.5 to 81.1) before surgery to 80.3 (range, 68.5 to 102) after surgery (*p* < 0.001).

**Figure 3:**
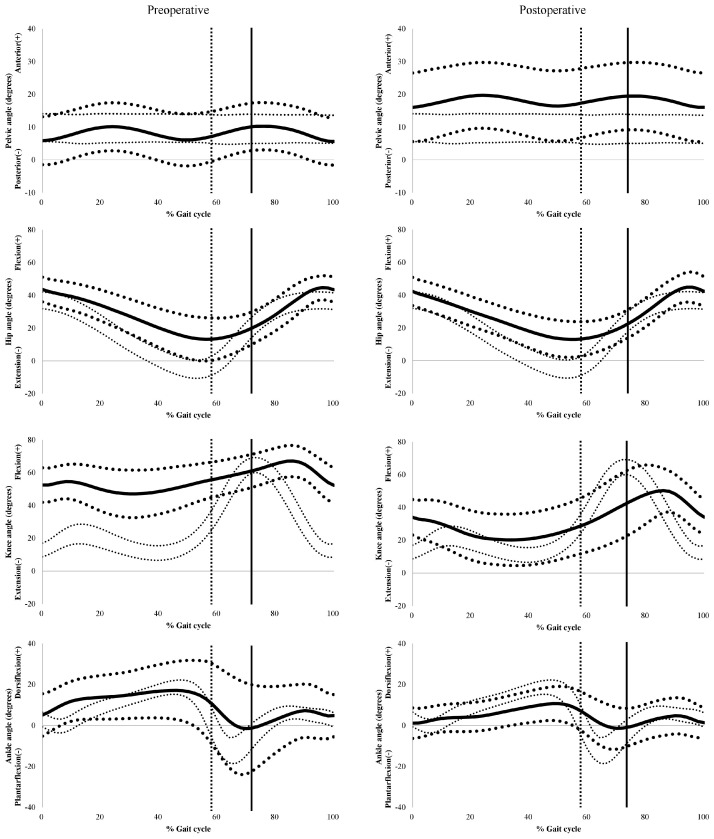
Preoperative and final follow-up sagittal plane kinematic graphs for the whole cohort. Bold solid lines indicate average values. Bold dotted lines represent one standard deviation. Fine dotted lines indicate the normal range.

Details on the results of gait analysis are presented in Table 3 and Figure 3. There was a significant increase of 9.9° in the mean anterior pelvic tilt after surgery (*p* < 0.001). The maximum anterior pelvic tilt also increased (*p* < 0.001), but the range of pelvic tilt in the sagittal plane decreased (*p* < 0.001). There were no significant changes in hip kinematics in the sagittal plane; however, there were significant improvements in the maximum knee extension during the stance phase (*p* < 0.001), knee flexion during the mid-stance (*p* < 0.001), range of knee flexion (=range of knee motion) (*p* < 0.001), and knee flexion during initial contact (*p* < 0.001). Although the range of knee motion during the gaiting increased, we observed that the maximum and the average knee flexion during the swing phase was decreased (*p* < 0.001) and the timing of peak knee flexion was delayed after the index operation, indicating a stiff knee gait pattern. The mean (*p* < 0.001) and the maximum (*p* < 0.001) ankle dorsiflexion during the stance phase, and dorsiflexion at the mid-stance (*p* < 0.001) significantly decreased after surgery, respectively.

In the subgroup analysis, there were no significant differences in the kinematic data before surgery between the groups, except the mean foot progression angle (Table 4). The mean foot progression angle in Group 2 was greater than that in Group 1 (*p* = 0.016), indicating the presence of a planovalgus foot deformity. After surgery no significant differences were found in all of the gait parameters between the groups, including the kinematics at the ankle and knee joints (Table 5).

**Figure 4:**
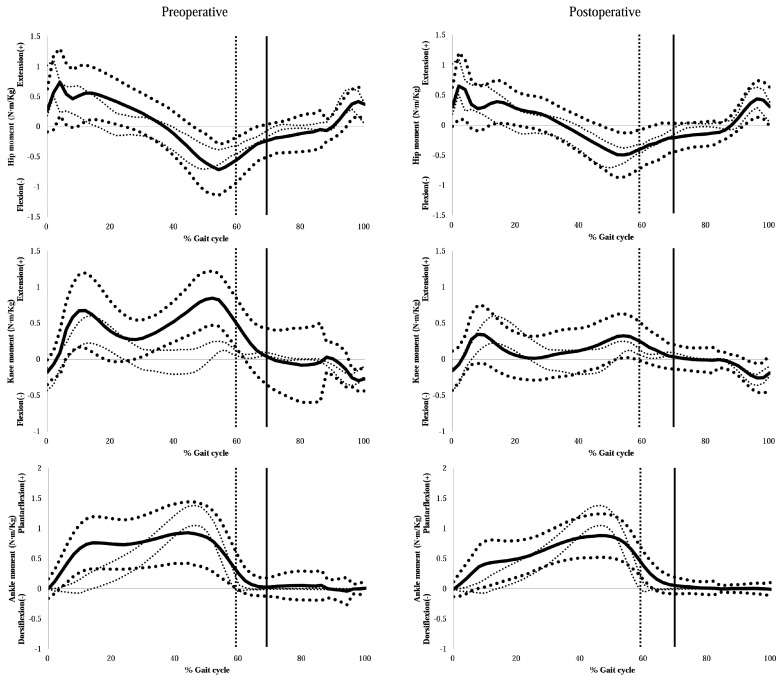
Preoperative and final follow-up sagittal plane kinetic graphs. Bold solid lines indicate average values. Bold dotted lines represent one standard deviation. Fine dotted lines indicate the normal range.

Kinetic analysis was performed in 19 patients (Table 6 and Figure 4). There were significant decreases in the maximum hip extensor moment (*p* = 0.003) at the early stance phase and the maximum hip flexor moment at the terminal stance phase (*p* < 0.001). Both the maximum and the average knee extensor moments decreased after surgery (*p* < 0.001), and the maximum ankle plantarflexion moment was also significantly decreased (*p* = 0.043).

## 4. Discussion

In the present study, we experienced significant improvements in both GDI and knee kinematics in the sagittal plane after the index operation. The amounts of these improvements were comparable to those previously reported in patients who had lesser degrees of contracture, treated by a DFEO [15,17,20,33]. This therefore indicates the effectiveness of our technique on the correction of a severe knee flexion contracture and a crouch gait.

During the last decade, DFEO has been suggested as an effective surgical option for correcting knee flexion contracture in patients with CP [14,15,16,17,18,19,20]. Additional PTA with either patellar tendon shortening or tibial tubercle advancement are used and known to be beneficial for retention of the muscle-tendon unit to correct extensor lag [7,21,22]. Recently, for the first time since the introduction of the procedure, Boyer et al. reported their long-term results of DFEO + PTA and compared their results with those of conventional treatments [20]. In this randomized control study, they found that gait parameters were most improved at short term for the DFEO + PTA participants, with a subsequent slight decline at long-term analysis [20]. Nevertheless, there still are concerns about postoperative neurologic complications caused by acute correction of the deformity and increased tension on the soft tissues structures posterior to the knee joint. The incidence rate of neurological complications after DFEO has been reported to be 3%–12% [15,23,34]. Stout et al. stated that the surgical indication for DFEO may be limited to patients with milder degrees of knee flexion deformity, and trimming of residual posterior bony prominence at the site of the osteotomy would be necessary to avoid stretching of the sciatic nerve [15]. Most of the literature reported that DFEO was performed in individuals with knee flexion contracture of less than 15–20 degrees [14,15,16,17,18,19,20]. There have been no prior studies which have investigated the effectiveness of a reconstructive surgery exclusively for ambulatory patients with severe knee flexion contracture, as observed in our series.

There were no significant changes in the temporospatial parameters and the GMFCS level did not change after surgery. Reduced knee extension during terminal swing is found to be associated with reduced hamstring length and velocity, and leads to a decrease in step length and a reduced walking speed [35]. Although our patients showed increased knee extension after surgery, there was no improvements in step length and/or walking speed. These results are in accordance with those of past studies on DFEO [18,36]. Our findings may be plausible, considering the “nature” of the study population at baseline: (1) 57.6% of our patients had previous trials of surgical intervention that eventually failed to improve gait function. Many had prior lengthenings of both the tendo-Achilles and the hamstrings, thereby weakening the two key muscle groups before the index operation had been performed; (2) 42.4% of the patients were in GMFCS level III in which impaired selective motor control is more frequently encountered [27]; (3) and finally, the severity of the crouch gait in our series, judged by mean knee flexion contracture of 38 degrees, leads us to redefine our expectations and think of the realistic surgical outcome of the index operation as being maintenance of the patient’s mobility level in the long term.

There are studies reporting an increase in the anterior pelvic tilt after DFEO [14,15,17,33]. Although the etiology for increased anterior pelvic tilt is still unclear, various underlying pathologies have been suggested: spasticity of hip flexor muscles [36,37,38]; weakness of the hip extensors or their elongated muscle length [12,39]; any imbalances between the hip flexors and extensors [40]; and weakness of the abdominal muscles [41]. In the present study, we also found that the anterior pelvic tilt increased significantly after surgery. We think that this deterioration may be related to both relative lengthening of the hamstrings due to femoral shortening and tightening of the quadriceps caused by PTA [15,19,36]. The inclusion of simultaneous hamstrings lengthening at the time of femoral osteotomy has been another issue [18,40]. Although hamstrings lengthening improved residual knee flexion contracture after DFEO, it led to an increase in anterior pelvic tilt instead [2,12,42]. A study using musculoskeletal modelling observed that only one third of patients with crouch gait had shortened hamstring muscles [39]. The length and velocity of hamstring muscles may increase after DFEO/PTA without concomitant hamstrings lengthening [40]. Our finding of an increased anterior pelvic tilt after the index operation implies that simultaneous hamstrings lengthening should not be performed, as the femoral shortening itself may indirectly lengthen the spastic hamstrings and thereby weaken the hip extensors and result in an increase of anterior pelvic tilt.

The effects of DFEO and/or PTA on the range of motion of the knee were inconsistent among studies [15,17,18,33,43]. However, we noticed in our study that the knee range of motion during walking was increased after the index operation, which was demonstrated on the kinematic study in the sagittal plane. This finding is in accordance with a study that investigated a two-stage femoral shortening and hamstring lengthening on patients with fixed knee contractures with an average of 22 degrees [25]. The range of motion of the knee was checked clinically and this may not be reflective of the changes seen whilst walking for the patients. When DFEO is performed for the correction of a severe knee flexion contracture, knee flexion is decreased by the degrees of the resected wedge-shaped bone but with alterations in the arc of knee motion and a corresponding reduction of the knee flexion angle [25]. Furthermore, a simple extension osteotomy for correction of a severe deformity creates an exaggerated posterior angulation at the distal femur and causes technical problems for fixation when using commercially available plates. Although this angulation may remodel or disappear over time in younger children, it may remain in adolescents and young adults. Additionally, as knee kinematics are influenced by the bony configuration of the distal femur and the femoral condyle offset ratio is positively correlated with the anterior tibial translation [44], the residual deformity after DFEO may lead to a decrease in the range of motion and a recurrence of knee flexion contracture. Even more worryingly, there may be a resultant tibio-femoral joint incongruity and a shift of the weight-bearing surface of the distal femur to the posterior femoral condyles during the mid-stance phase of gait. The minimal increase in pPDFA noticed at the final follow-up implies that the alignment of the distal femoral articular surface has been preserved.

Our results suggested that the crouch gait deformity may be converted into a stiff knee gait after the index operation. It has been noted that there is a potential risk for the development of a stiff knee gait due to quadriceps tightness from a PTA [14,19,43,44]. A study using generic musculoskeletal modelling postulated that the quadriceps undergoes substantial shortening with DFEO, and that this can be compensated by performing a simultaneous PTA [24]. Contrary to our belief that DFSO has more of a tension-relieving effect on the quadriceps than DFEO, the degrees of maximum knee flexion in swing phase decreased and the timing of peak knee flexion was delayed. We are not aware of the mechanisms by which the stiff knee gait becomes apparent after surgery; however, there is a possibility that the unresolved spasticity of the hamstrings and “relative tightening” of the quadriceps resulting from a PTA may be responsible for the development of stiff knee gait. Despite this, we noticed a postoperative increase in the range of knee motion during the swing phase, which is beneficial in the pre-positioning of the foot.

We found significant changes in the ankle kinematics in the whole cohort. In stance phase, there was a decrease in the mean and the maximum dorsiflexion after surgery. According to the concept of ankle plantarflexion and knee extension coupling, improved knee flexion contracture leads to a decrease in the ankle dorsiflexion angle. However, previous studies on the results of DFEO with or without PTA show inconsistent results [17,19,33]. This discrepancy may be due to the various additional surgeries performed simultaneously in these studies. In our series, a concurrent reconstructive foot surgery and plantarflexor release were performed in 11 patients. A subgroup analysis was completed in order to examine the effects of making a plantigrade foot on the concept of the ankle plantarflexion/knee extension couple. However, there were no significant differences in the hip, knee, and ankle kinematics between the groups, before and after surgery, and it did not seem that the foot reconstruction influenced the results of the index surgery.

Our postoperative kinetic analysis, performed in 19 subjects, showed significant improvement at the hip and knee joints. The maximum hip extensor and flexor moments, and the maximum and average knee extensor moments were decreased after surgery. These changes may be due to the improved knee flexion contracture which has shifted the ground reaction forces closer to the centers of the hip and knee joints, thereby reducing the demands on the antigravity support muscles. We found no improvement in ankle kinetics. The randomized controlled study of Boyer et al. noted that one third of the patients continued to show a crouch gait in the long term and all patients slightly regressed towards their baseline status due to persistent plantar flexor weakness [20]. Although knee and ankle kinematics improved in our series, the long-term outcomes might be similar to those of Boyer et al. [20] as the ankle plantar flexion moments were found to be decreased despite the correction of the foot deformity. Our findings suggested that many of our patients already had weakened plantarflexors due to the presence of their long-standing planovalgus foot deformity. Furthermore, we believe that the performance of a gastrosoleus lengthening for the equinus deformity, which becomes evident right after the correction of pes planovalgus, may further weaken the triceps power.

Several limitations to this study should be mentioned. First, the study design was retrospective and, as such, it may be affected by potential bias as it lacks a control group. Second, DFSO was performed in the context of single-event multilevel surgery. Although DFSO is mainly aimed at correcting flexed knee gait, the changes in the kinematic and kinetic variables may have been influenced by the concomitant procedures. Third, we focused on changes in the gait analysis and in the radiological evaluation but did not investigate changes in the patient’s quality of life. And finally, although we performed a subgroup analysis to evaluate the function of the foot, its power was low due to the small sample size.

## 5. Conclusions

We conclude that the combined procedure of DFSO and PTA is an effective and safe surgical method to treat severe knee flexion contracture and crouch gait in CP. It restores sagittal alignment of the knee joint and improved many of clinical, radiological, and gait parameters. However, an increased anterior pelvic tilt and the development of a stiff knee gait remains as future issues that need to be investigated further through the use of patient reported outcome measures.

## Figures and Tables

**Table 1:** The lists of previous and simultaneous surgeries.

Types of Surgery	Previous Surgery	Simultaneous Surgery
Osseous surgery		
Tibial derotation osteotomy	2	8
Reconstruction of lateral/medial column of the foot	11	11
Soft-tissue release		
Psoas	8	2
Adductor	10	13
Hamstring	17	0
Plantarflexors	16	11

**Table 2:** Outcome Measures.

	Preoperative	Final Follow-Up	*p*-Value
Physical examination			
Knee flexion contracture (°)	38 ± 6	12 ± 7	<0.001
Popliteal angle (°)	68 ± 16	46 ± 11	<0.001
Radiographic parameters			
pPDFA (°)	86.7 ± 3.6	88.1 ± 4.8	0.014
Koshino index	1.56 ± 0.17	1.24 ± 0.17	<0.001
Koshino index (z-score)	2.3 ± 1.8	−0.7 ± 1.9	<0.001
Gait parameters			
Walking speed (cm/second)	54 ± 33	48 ± 35	0.288
Step length (cm)	35 ± 14	32 ± 15	0.232
Cadence (steps/min)	86 ± 33	80 ± 34	0.253

All values are expressed as the means ± SD. pPDFA, physeal posterior distal femoral angle.

**Table 3:** Preoperative and final follow-up kinematic results for the whole cohort.

	Preoperative	Final Follow-Up	*p*-Value
Pelvis			
Maximum pelvic tilt (°)	12.3 ± 7.0	21.4 ± 10.0	<0.001
Average pelvic tilt (°)	8.2 ± 7.1	18.1 ± 10.3	<0.001
Range of pelvic tilt (°)	8.6 ± 3.3	7.0 ± 2.5	<0.001
Hip			
Maximum hip extension in stance (°)	11.1 ± 11.4	11.7 ± 10.5	0.539
Maximum hip flexion in swing (°)	46.4 ± 7.7	46.1 ± 9.3	0.789
Knee			
Maximum knee extension in stance (°)	44.7 ± 13.3	18.3 ± 15.3	<0.001
Knee flexion at mid-stance (°)	47.2 ± 14.6	20.1 ± 16.2	<0.001
Maximum knee flexion in swing (°)	70.1 ± 8.9	56.0 ± 11.3	<0.001
Range of knee flexion (°)	25.4 ± 9.7	37.7 ± 10.9	<0.001
Knee flexion at initial contact (°)	52.4 ± 10.7	33.4 ± 11.6	<0.001
Ankle			
Mean dorsiflexion in stance (°)	12.2 ± 12.8	5.6 ± 6.7	<0.001
Maximum dorsiflexion in stance (°)	20.7 ± 13.3	12.5 ± 7.4	<0.001
Mid-stance dorsiflexion (°)	16.1 ± 12.7	7.7 ± 7.3	<0.001
Maximum plantarflexion in swing (°)	−8.7 ± 17.4	−5.2 ± 9.1	0.072
Maximum dorsiflexion in swing (°)	8.9 ± 12.5	6.3 ± 8.5	0.035

All values are expressed as the means ± SD.

**Table 4:** Comparisons of preoperative kinematic parameters between the Groups 1 and 2.

	Group 1 (*n* = 22)	Group 2 (*n* = 11)	*p*-Value
Pelvis			
Maximum pelvic tilt (°)	11.6 ± 5.5	13.9 ± 9.4	0.217
Average pelvic tilt (°)	7.2 ± 5.7	10.4 ± 9.1	0.100
Range of pelvic tilt (°)	8.8 ± 3.0	8.0 ± 3.8	0.348
Hip			
Maximum hip extension in stance (°)	9.3 ± 11.6	15.1 ± 10.3	0.050
Maximum hip flexion in swing (°)	46.4 ± 6.6	46.3 ± 9.8	0.955
Knee			
Maximum knee extension in stance (°)	43.3 ± 13.5	47.7 ± 12.5	0.208
Knee flexion at mid-stance (°)	45.8 ± 15.0	50.4 ± 13.5	0.224
Maximum knee flexion in swing (°)	70.2 ± 8.8	69.7 ± 9.4	0.849
Range of knee flexion (°)	26.9 ± 9.5	22.0 ± 9.4	0.060
Knee flexion at initial contact (°)	51.0 ± 10.8	55.5 ± 9.9	0.109
Ankle			
Mean dorsiflexion in stance (°)	10.9 ± 12.5	15.1 ± 13.3	0.223
Maximum dorsiflexion in stance (°)	19.8 ± 13.5	22.8 ± 13.0	0.404
Mid-stance dorsiflexion (°)	15.0 ± 12.4	18.4 ± 13.3	0.328
Maximum plantarflexion in swing (°)	−10.1 ± 16.5	−4.5 ± 18.9	0.235
Maximum dorsiflexion in swing (°)	8.2 ± 11.8	10.7 ± 14.1	0.465
Mean foot progression angle (°)	1.2 ± 19.11	12.9 ± 16.2	0.016

All values are expressed as the means ± SD.

**Table 5:** Comparisons of postoperative kinematic parameters between the Groups 1 and 2.

	Group 1 (*n* = 22)	Group 2 (*n* = 11)	*p*-Value
Pelvis			
Maximum pelvic tilt (°)	20.0 ± 9.7	24.4 ± 10.3	0.255
Average pelvic tilt (°)	16.5 ± 9.9	21.4 ± 10.7	0.218
Range of pelvic tilt (°)	7.4 ± 2.4	6.3 ± 2.7	0.283
Hip			
Maximum hip extension in stance (°)	9.5 ± 10.1	16.6 ± 9.7	0.057
Maximum hip flexion in swing (°)	45.0 ± 9.9	48.6 ± 7.4	0.299
Knee			
Maximum knee extension in stance (°)	19.2 ± 15.9	16.1 ± 14.2	0.590
Knee flexion at mid-stance (°)	20.6 ± 16.6	19.1 ± 15.6	0.800
Maximum knee flexion in swing (°)	56.2 ± 12.1	55.5 ± 9.8	0.853
Range of knee flexion (°)	37.0 ± 10.4	39.3 ± 12.0	0.543
Knee flexion at initial contact (°)	32.8 ± 13.0	34.6 ± 7.7	0.676
Ankle			
Mean dorsiflexion in stance (°)	5.9 ± 7.0	4.9 ± 6.1	0.688
Maximum dorsiflexion in stance (°)	13.1 ± 7.4	11.0 ± 7.2	0.407
Mid-stance dorsiflexion (°)	8.0 ± 7.6	7.1 ± 7.0	0.729
Maximum plantarflexion in swing (°)	-6.2 ± 9.7	-2.9 ± 7.5	0.303
Maximum dorsiflexion in swing (°)	6.4 ± 9.0	6.2 ± 7.6	0.940
Mean foot progression angle (°)	8.7± 10.6	8.7 ± 11.2	0.994

All values are expressed as the means ± SD.

**Table 6:** Preoperative and final follow-up kinetic variables for the whole cohort.

	Preoperative	Final Follow-Up	*p*-Value
Hip extension moment (N∙m/Kg)			
Maximum	1.13 ± 0.46	0.91 ± 0.48	0.003
Minimum (=maximum flexor moment)	−0.86 ± 0.36	−0.62 ± 0.31	<0.001
Average	0.21 ± 0.16	0.12 ± 0.17	0.794
Knee extension moment (N∙m/Kg)			
Maximum	0.99 ± 0.41	0.56 ± 0.33	<0.001
Minimum (=maximum flexor moment)	−0.41 ± 0.49	−0.37 ± 0.20	0.621
Average	0.28 ± 0.24	0.08 ± 0.19	<0.001
Ankle plantarflexion moment (N∙m/Kg)			
Maximum	1.15 ± 0.37	1.00 ± 0.36	0.043
Minimum	−0.12 ± 0.31	−0.06 ± 0.10	0.228
Average	0.43 ± 0.21	0.37 ± 0.15	0.151

All values are expressed as the means ± SD.
